# Quantifying the impact of positive stress on companies from online employee reviews

**DOI:** 10.1038/s41598-022-26796-6

**Published:** 2023-01-28

**Authors:** Sanja Šćepanović, Marios Constantinides, Daniele Quercia, Seunghyun Kim

**Affiliations:** 1 Nokia Bell Labs, Cambridge, UK; 2 CUSP, Kings College London, London, UK; 3 Georgia Tech, Atlanta, USA

**Keywords:** Computer science, Complex networks

## Abstract

Workplace stress is often considered to be negative, yet lab studies on individuals suggest that not all stress is bad. There are two types of stress: *distress* refers to harmful stimuli, while *eustress* refers to healthy, euphoric stimuli that create a sense of fulfillment and achievement. Telling the two types of stress apart is challenging, let alone quantifying their impact across corporations. By leveraging a dataset of 440 K reviews about S &P 500 companies published during twelve successive years, we developed a deep learning framework to extract stress mentions from these reviews. We proposed a new methodology that places each company on a stress-by-rating quadrant (based on its overall stress score and overall rating on the site), and accordingly scores the company to be, on average, either a *low stress*, *passive*, *negative stress*, or *positive stress* company. We found that (former) employees of positive stress companies tended to describe high-growth and collaborative workplaces in their reviews, and that such companies’ stock evaluations grew, on average, 5.1 times in 10 years (2009–2019) as opposed to the companies of the other three stress types that grew, on average, 3.7 times in the same time period. We also found that the four stress scores aggregated every year—from 2008 to 2020 —closely followed the unemployment rate in the U.S.: a year of positive stress (2008) was rapidly followed by several years of negative stress (2009–2015), which peaked during the Great Recession (2009–2011). These results suggest that automated analyses of the language used by employees on corporate social-networking tools offer yet another way of tracking workplace stress, allowing quantification of its impact on corporations.

## Introduction

According to the American Institute of Stress, 40% of workers consider their jobs to be stressful; a number that has significantly increased during the COVID-19 pandemic^1^. The World Health Organization treats stress as the number one health threat in the U.S., with more than 60% of doctor visits being due to a stress-related issue^2^. Workplace stress is often linked to lower motivation, poor performance, and decline in employees’ well-being^3^, while it is estimated to amount to 190 billions in healthcare costs in the U.S. alone^4^. To currently track how its employees deal with stress, a large company would typically recruit consultants who would then administer surveys tailored to the company’s situation, which typically end up being costly^5^, and restricted to a limited pool of self-selected participants^6–8^. The current situation points to the need of more research.

Stress is defined as “a set of physical and psychological responses to external conditions or influences, known as stressors”^9^. According to Lazarus^10^, “any change, either good (eustress) or bad (distress), is stressful, and whether it is a positive or a negative change, the physiological response is the same.” To cope with workplace stress, an employee has to cognitively acknowledge that a situation causes stress before even attempting to manage it^11^. Kobasa’s framework of psychological hardiness offers three main categories of coping strategies^12^: *commitment* (having an active involvement in one’s own work with a sense of purpose), *control* (believing and acting instead of feeling helpless in front of adversity), and *challenge* (believing that change could be a source of improvement). Kobasa posited that these categories could help individuals face challenges and, as such, individuals could turn stressful events into opportunities for personal growth^12^. However, despite having explored the relation between stress and job performance for decades, researchers have not yet established whether stress and performance are in a negative linear relation, a positive linear relation, or an inverted-U relation^13^.

To tackle this gap, we draw upon two streams of previous literature, that is, literature about stress in the corporate context, and literature on how to gauge stress from online data. In the literature about stress in the corporate context, stress is often being portrayed as negative^14^; and as a leading cause of death, poor work performance, and diminishing well-being^3^. More recently, however, researchers have advocated that there exist another type of stress: *positive stress*. The idea is that whether stress is positive or negative depends on how an individual reacts to a stressor^15^. *‘One’s stress mindset can be conceptualized as the extent to which one holds the belief that stress has enhancing consequences for various stress-related outcomes such as performance and productivity, health and well-being, and learning and growth, or holds the belief that stress has debilitating consequences for those outcomes *^15^’. Of prime importance is to distinguish appraisal from stress mindset. Stress mindset describes the evaluation of the nature of stress itself as positive or negative (i.e., enhancing or debilitating)^15^, whereas appraisal is about the evaluation of a particular stressor as more or less stressful^16^. For example, one may appraise a difficult task as highly stressful and have a stress debilitating mindset, which, in turn, leads the individual to experience the situation as draining (negative stress). By contrast, another individual may again consider the task as highly stressful but have a stress enhancing mindset, leading the individual to experience the situation as an opportunity for growth and development (positive stress). While stress is often linked to depression^17,18^, several accounts posit that certain stressful experiences may fundamentally change individuals for the better—a phenomenon referred to as stress-related growth^15^. The experience of stress can enhance the development of mental toughness, greater appreciation for life, and an increased sense of meaningfulness^19,20^. However, as Crum et al.^15^ pointed out, these conflicting findings in the stress literature suggest a nuanced view of stress. A view that recognizes the debilitating nature of stress on health and performance, but can also account for its enhancing nature in specific circumstances. We hypothesized that the presence of both positive and negative stress can be measured from digital data based on previous literature that has done just that with different techniques upon different datasets. Guntuku et al.^21^ used the Linguistic Inquiry and Word Count (LIWC)^22^ dictionary’s features (e.g., topical categories, emotions, parts-of-speech) to predict stress of social media (Facebook and Twitter) users. Saha and De Choudhury^23^ did a similar study but on Reddit and did so in conjunction with gun violence events, and found specific stress markers to be associated with linguistic changes about *“higher self pre-occupation and death-related discussion.”* Similar to our study, Vedant et al.^24^ showed that the use of language in employee reviews can be used to operationalize organizational culture: the collection of values, expectations, and practices that guide and inform employees’ actions within a company.

Based on these preliminary findings, we hypothesized that workplace stress is reflected in company reviews. To explore this hypothesis, we placed companies on a 2x2 coordinate system, based on their overall stress scores and overall ratings on the company review site. This stress-by-rating quadrant effectively divided companies in four stress types that we termed low stress, passive, negative stress, and positive stress (Table 1 shows example reviews of companies of each stress type). Low stress companies enjoy high overall ratings and low stress scores. These are usually established organizations that offer workplace flexibility, good pay, and bonuses. Passive companies are characterized by low overall ratings and a small proportion of posts mentioning stress. They tend to have high turnover, due to repetitive workload and non-motivated employees. Negative stress companies are characterized by low overall ratings and a high proportion of posts mentioning stress. Employees of these companies are particularly unhappy as, in addition to the unsatisfactory conditions, they also experience high pressure. Finally, positive stress companies enjoy high overall ratings but also high stress scores. These tend to be inspiring, reputable workplaces that attract employees because of the collaborative atmosphere and career prospects despite the pressure employees are subject to. The project website for our study is found on https://social-dynamics.net/positive-stress.

### Data

After obtaining the U.S. unemployment rates between 2008 and 2020 from the U.S. Bureau of Labor Statistics^25^ and the S &P 500 stock market data (including the 500 large capital U.S. companies with a cumulative market capitalization to be around 70-80% of the total in the country) from the Yahoo Finance portal^26^, we matched that data with our company reviews. More specifically, we obtained 440K geo-referenced posts on Glassdoor (https://www.glassdoor.com), a popular company reviewing site about the S &P 500 companies published during twelve successive years, from 2008 to 2020. On this site, current and, more likely, former employees of companies write reviews about their own corporate experience, ranging from job interviews to salaries to workplace culture. The site provided an overall rating of each company based on employees’ reviews. As of 2021, there are 50M monthly visitors on the platform, and 70M reviews about 1.3M companies. To ensure quality reviews, the site employs the following three mechanisms. First, an automatic (proprietary) and manual content moderation system is paired with the possibility for users to flag content. Such a combined system partly prevents fake reviews (e.g., a company unfairly forcing employees to leave positive reviews). Second, every user wanting to browse others’ content has to take the time to write one review. This requirement encourages the presence of neutral reviews and partly prevents the so-called *non-response bias*, a situation in which the opinions of respondents are very different from those of non-respondents. Third, the site allows for a maximum of one review per employee per company per year, preventing any employee from contributing a disproportionate number of reviews, and, in so doing, discouraging *sampling bias*, a situation in which the opinions of some members are more represented in the data than those of others.

**Table 1:** Example reviews of companies of each stress type.

]Stress Type	Review excerpt
Low stress	My company walks its talk. It *[the company]* takes care of customers and employees.
Negative stress	There is a feeling of scarcity due to the constant reorganizations, pressure, and surprise layoffs. *[...]* You could imagine how toxic the environment is.
Passive stress	There is no regard for how the remaining work will get done, just how the bottom line looks at that moment in time. People are not treated as respected contributors to the organization. *[...]* This is a very unstable, unhealthy, volatile, stressed out environment, with incredibly poor leadership.
Positive stress	You have to be a very driven and self-motivated person to be successful here. If you are willing to commit and put in the extra effort and hard work, it will be extremely worth it. *[...]* Every day is very busy and it can be stressful at times but its all worth it!.

## Methods

**Figure 1:**
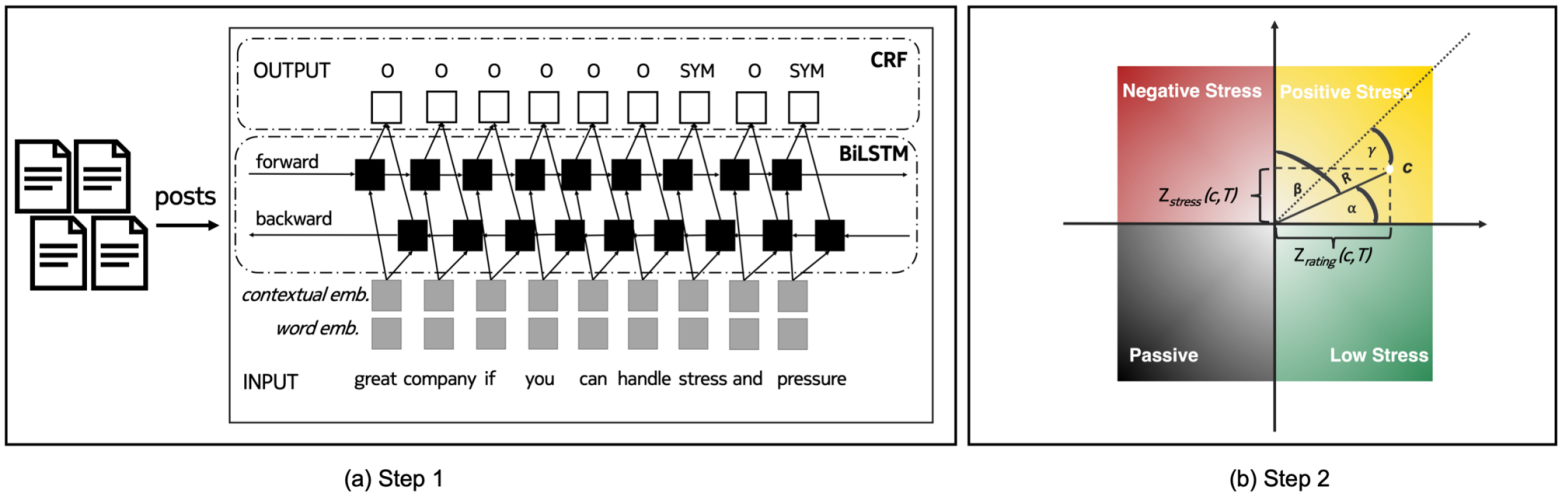
Placing companies on a stress-by-rating quadrant by detecting stress mentions in reviews about a company using a state-of-the-art NLP deep-learning framework (*Step 1*), placing the company in the rating-by-stress quadrant, and computing its association with its stress type (i.e., with its quadrant) (*Step 2*). To see how the association is computed, consider company *c* shown in *(b)* to be of positive stress. *c* is placed according to its $z_{rating}(c,T)$ along the *x*-axis, and to its $z_{stress}(c,T)$ along the *y*-axis. *R* is the radius from the center to *c*’s point; $\alpha$ is the angle between the radius line and the *x*-axis; $\beta$ is the angle between the radius line and the *y*-axis; and $\gamma$ is the angle between the radius line and the diagonal shown as a dotted line. The function *f*(*c*, *s*, *T*) combines *R*, $\alpha$, $\beta$, and $\gamma$, and accordingly scores *c* to have a high association weight with positive stress *s* during period *T* (darker shades of colors), as *c* is close to the quadrant’s diagonal, and distant from the intersection point.

We extracted mentions related to stress using an NLP deep-learning tool, which is trained to extract medical conditions from free-form text (Fig. 1a). We then designed a new methodology that placed the 500 S &P companies on a stress-by-rating quadrant based on their overall ratings on the reviewing site on one axis, and the presence of mentions related to stress in their reviews on the other axis (Fig. 1b). In so doing, we classified each company to be, on average, either a *low stress*, *passive*, *negative stress*, or *positive stress* company. We finally computed each company’s strength of membership to its quadrant depending on whether the company is placed both close to the diagonal and far from the (0,0) intersection point. The function *f*(*c*, *s*, *T*), which is expressed in Eq. (1) and graphically depicted in Fig. 1b, assigns a higher weight to company *c* of stress type *s*, if *c* is both closer to the quadrant’s diagonal (i.e., it is farther from the remaining quadrants) and more distant from the two axes’ intersection (i.e., it has higher absolute overall rating and stress score values). We call *f*(*c*, *s*, *T*) to be company *c*’s association with stress type *s* during *T* since the higher its value, the more *c* is associated with stress type *s* during *T*.

*Extracting stress mentions from posts* To extract stress mentions, we used the MedDL entity extraction module^27^ (the left rectangle in Figure 1 (a)). MedDL uses *contextual embeddings* and a *deep BiLSTM-CRF sequence labeling architecture* (we used the default parameters as specified in^27^). The model was pre-trained and evaluated on a labeled dataset of Reddit posts called MedRed^27^. The MedRed dataset was split into train (50%), dev (25%), and test (25%) sets. We evalued MedDL using the strict and relaxed *F*1-scores, two commonly used performance metrics for entity extraction models. The *strict F1-score* counts only the *exact* matches with the true labels as correct, while the *relaxed F1-score* takes into accoun the partially matching extracted entities. We provide formulae for the two scores in *Supplementary Information* (SI Eq. (1)). MedDL was compared against two well-known entity extraction tools: MetaMap (a well-established tool^28^ and a de-facto baseline method for NLP studies related to health^29^) and TaggerOne (a machine learning tool using semi-Markov models and a medical lexicon^30^). The MedDL method achieved a strict/relaxed *F*1-score of .71/.85 when extracting symptoms (Figure S4), outperforming both MetaMap and TaggerOne by a large margin (the two have *F*1-scores of .17/.48 and .31/.58, respectively). Furthermore, MedDL has shown generalizability when applied on dataset (e.g., dream reports^31^) different than those it was trained on (i.e., Reddit data).

**Table 2:** Top15 most frequent stress-related mentions identified on a company review site, and their frequency counts.

Condition related to stress	# mentions	Example mention
Stress	3473	*“Great company to work for, if you can handle stress.”*
High stress	710	*“High stress work environment, long work hours.”*
Pressure	447	*“a lot of pressure to get things done.”*
Burnout	277	*“[...], the ones who made the cut to stay are suffering from burnout.”*
Understaffing	99	*“Somewhat job stability due to understaffing.”*
Heavy workload	58	*“Lack of work/life balance, extremely heavy workload.”*
Exhaustion	58	*“You will be pushed to the point of exhaustion [...].”*
Stress levels	57	*“[...] stress levels peak insanely when the store manager [...].”*
Overworked	45	*“At times, you can feel overworked and undervalued.”*
Tension	38	*“There’s a lot of tension between coworkers because of commission.”*
High workload	38	*“[...] seeing many large set-backs which cause very high workload”*
Extreme stress	33	*“Beware: extreme stress and pressure.”*
Mental stress	23	*“[...] ends up giving you a lot of mental stress.”*
Overload	17	*“No work life balance [...], overloaded and benefits are not good.”*
Pressure to perform	9	*“[...] a lot of pressure to perform, long working hours”*

We extracted stress mentions in three steps (further detailed in *Supplementary Information*). First, we detected over 21K posts that mentioned over 5K unique medical conditions. Most frequent medical conditions identified include *stress*, *pain*, *headache*, and *depression*. Second, we inspected the $\text {top 200}$ most mentioned conditions and manually selected 31 of them that specifically reflect workplace stress ($\text {top 15}$ are shown in Table 2). Third, we extracted all reviews mentioning any of the 31 conditions. This resulted in 7, 338 posts related to stress, which accounted for $1\%$ of all posts. Despite this seemingly low number of posts, when aggregated, these posts returned statistically significant results for our metrics, which are described next.

*Associating stress types with companies* We placed each S &P 500 company on a stress-by-rating quadrant. More specifically, for each company *c*, we computed its average review rating and its stress score:

$$\begin{aligned} {\text{rating}} (c,T) & = c^{\prime}s\,average\;review\;rating\;during\,T, \\ stress(c,T) & = \frac{{\# {\mkern 1mu} {\mkern 1mu} c^{\prime}sposts\;related\;to\;stress\;during{\mkern 1mu} {\mkern 1mu} T}}{{total\;\# \;c^{\prime}sposts\;during\;T}}. \\ \end{aligned}$$

where *T* is set to initially include all the years under study (2009–2019). To then ease comparability, we *z*-scored these two values:

$$\begin{aligned} z_{rating}(c,T)&= \frac{rating(c,T) - \mu _{rating}(T)}{\sigma _{rating}(T)}, \\ z_{stress}(c,T)&= \frac{stress(c,T) - \mu _{stress}(T)}{\sigma _{stress}(T)}. \end{aligned}$$

where $\mu _{rating}(T)$ and $\sigma _{rating}(T)$ are the average and standard deviation of the review ratings for all companies (regardless of their stress types) during the whole period *T* (readily available on the company review site), and $\mu _{stress}(T)$ and $\sigma _{stress}(T)$ are the average and standard deviation of the stress scores for all companies during the whole period *T*.

Each S &P 500 company was assigned to one of the four quadrants based on the signs of their two z-scores (Fig. 1b). For example, a company *c* with a negative $z_{rating}(c,T)$ and a positive $z_{stress} (c,T)$ would be placed in the negative stress quadrant, while a company with a positive $z_{rating}(c,T)$ and a positive $z_{stress} (c,T)$ would be placed in the positive stress quadrant. The resulting quadrants are consequently four:

- *Low Stress companies* These enjoy high overall ratings and low stress scores. Their employees tended to think very positively about their workplace experience with comparatively fewer posts mentioning stress conditions.
- *Passive companies* These are characterized by low overall ratings and a small proportion of posts mentioning stress. Their employees were mostly not satisfied with their jobs, but they also showed comparatively fewer signs of stress in their reviews.
- *Negative stress companies* These are characterized by high stress scores and low overall ratings. Their employees mentioned stress conditions, while also scoring their workplace experience low.
- *Positive stress companies* These enjoy high ratings despite high stress scores. Their employees mentioned stress yet did so in the context of high-pressure and highly rewarding work environments.

Once a company *c* is placed in its quadrant (i.e., associated with its stress type *s*), we needed to estimate its association with this quadrant, i.e., with *s*. For example, company *c* with ($z_{rating} (c,T), z_{stress}(c,T)$) equal to (3,3) is more strongly associated with *positive stress*, than what a company with (0.5, 0.5) would be. To estimate *c*’s association with *s*, we combined *c*’s two *z*-scores concerning review rating and stress score as follows (and as depicted in Fig. 1b):

1 $$\begin{aligned} f(c,s, T)&=\left\{ \begin{array}{@{}ll@{}} l ( z_{rating}(c,T), z_{stress}(c,T) ) = R / (\gamma + \pi ), &{} \text {if}\ c \in s \text { during } T;\\ 0, &{} \text {if}\ c \notin s \text {, or } c \text { has no review during } T; \end{array}\right. \nonumber \\ \text {where:} \nonumber \\ R&= \sqrt{ z_{rating}(c,T) {}^2 + z_{stress}{}(c,T)^2}, \nonumber \\ \gamma&= max ( (\alpha - \pi / 4), (\beta - \pi / 4)), \quad \quad \nonumber \\ \alpha&= arccos(|z_{rating}(c,T){})| / R), \quad \quad \quad \quad \quad \nonumber \\ \beta&= arccos(|z_{stress}(c,T){})| / R). \end{aligned}$$

where *T* is initially set to include all the years under study, from 2009 to 2019. To ease understanding of the above formula, consider that function *l*, on input of the two *z*-scores (i.e., the company’s two coordinates in the quadrant), computes the extent to which company *c* is on the diagonal and far from the (0,0) intersection point (Fig. 1b). It gives higher weights to companies that are both closer to the quadrant’s diagonal (i.e., which are farthest from the remaining quadrants) and more distant from the axes’ intersection (i.e., which have higher absolute rating/stress score values).

*Computing stress scores over the years* For each year *y*, we quantified the amount of a given stress type *s* expressed in the posts produced in that year. More specifically, we computed:

2 $$\begin{aligned} m{(s,y)} = \sum _{c \in s} f(c,s,y) \times w{(c,y,s)}, \end{aligned}$$

For all the companies of stress type *s*, we summed each company’s association *f*(*c*, *s*, *y*) with *s* during year *y* weighted by the presence of posts about the company during *y* (giving higher weights to companies whose employees contributed more reviews in that year):

3 $${\text{w(c,y,s)}} = \left\{ {\begin{array}{*{20}c} {\frac{{\# \;c^{\prime}\,sposts\;in\;year\,y}}{{total\;\#\, posts\;in\;year\;y}},} & {{\text{if}}\;c \in s\,in\,year\,y;} \\ {0,} & {{\text{if}}\;c\,{\text{has }}\;{\text{no}}\;{\text{ reviews}}\;{\text{ in}}\;{\text{ year}}\;y.} \\ \end{array} } \right.{\text{ }}$$

*Associating topical categories with stress types* To identify relevant words for each stress type, we run BERTopic^32^, which is a state-of-the-art topic modeling algorithm. A topic modeling algorithm is an unsupervised technique to extract topics that appear frequently in a piece of text (in our case, a post). The algorithm works in four sequential steps:

1. converts each post into a 512-dimensional vector (called embedding) of numerical values using a pre-trained BERT-based sentence transformer^33^ (in our case, we used the default model, that is, the “paraphrase-MiniLM-L6-v2”). BERT (Bidirectional Encoder Representations from Transformers) is a state-of-the-art transformer-based machine learning technique for natural language processing (NLP), which takes into account the context of each word.
2. reduces dimensionality using UMAP^34^ (or Unification Map) for every embedding, as many clustering algorithms handle high dimensionality poorly. UMAP is arguably the best performing dimensionality reduction algorithm as it keeps significant portion of the high-dimensional structure in lower dimensionality.
3. uses HDBSCAN^35^ for clustering with the “UMAP” embeddings, resulting in similar posts being clustered together. HDBSCAN is a density-based algorithm that works well with UMAP as the structure is preserved in a lower-dimensional space. Additionally, HDBSCAN does not force data points to clusters as it considers them outliers.
4. identifies keywords using the c-TF-IDF^32^ score (Eq. 4), and using that score, derives topics from the identified keywords. To create a topic representation, we took the top 3 keywords per topic based on their c-TF-IDF scores. The higher the score, the more representative is as the score is a proxy of information density.
  4 $$\begin{aligned} \text {c-TF-IDF}_{l} = \frac{k_{l}}{o_{l}} \times \frac{p}{\sum _{j}^{q}k_{j}} \end{aligned}$$
  where the frequency of each keyword *k* is extracted for each topic *l* and divided by the total number of keywords *o*. The total, unjoined, number of posts *p* is divided by the total frequency of keyword *k* across all topics *q*.

### Analysis plan

Our analysis plan unfolded in three steps. First, as an initial validation step, we ascertained that stress was paraphrased in a company’s reviews differently according to the company’s stress type. Second, we tested whether the evolution of each stress score over the years tallied with large-scale exogenous events such as the Great Recession. Third, we tested that a company’s stress type is partly associated with its stock growth.

## Results

### Topics discussed in reviews of companies of different stress types

To ascertain whether the content of the reviews captured aspects specific to the four stress types, we identified the top relevant words for each type by running a topic modeling algorithm called BERTopic^32^, and did so on four distinct sets of reviews: each set contained reviews of all the companies of a given stress type. This algorithm found the emergent topics in the four sets, and Table 3 lists the the top three words for each topic. The top10 topics for each quadrant are statistically associated with the quadrant. That is, based on chi-square tests, each topic *l* associated with quadrant *s*: has frequency in *s* always above zero (is dependent on *s*), and is independent of any quadrant other than *s*. As detailed in *Supplementary Information*, by inspecting these groups of words and corresponding representative reviews, six annotators identified the emergence of three workplace themes:

- *Career drivers* (first set of rows in Table 3). Negative stress companies were associated with words such as ‘overtime’, ‘mandatory, ‘shifts’, and the typical workplace described in the reviews, according to our annotators, was characterized by considerable emotional pressure. On the other hand, passive companies were associated with words such as ‘vacation’, ‘pto’, and ‘vacation/sick’, and the corresponding reviews tended to deflect from the day-to-day work and focus on activities outside work such as vacation and time off. Low stress companies were associated with words such as ‘scheduling’, ‘flexibility’, and ‘autonomy’, and the typical workplace described in the reviews was one in which employees cherished their sense of control over their work. Finally, positive stress companies were associated with words such as ‘teamwork’, ‘supportive’, and ‘collaborative’, and the typical workplace in the reviews was one with a collaborative and supportive culture.
- *Industry or benefits* (second set of rows in Table 3). Negative stress companies were associated with words such as ‘discounts’, ‘sale’, ‘coupons’, while positive stress companies were associated with words such as ‘gain’, ‘billions’, and ‘software’. Their reviews were effectively mentioning the industry sectors they referred to: Consumer Discretionary (e.g., retail shops) for the reviews of negative stress companies, and Information Technology for those of positive stress ones. On the other hand, passive companies were associated with words such as ‘insurance’, ‘espp’, and ‘hsa’, and, similarly, low stress ones with words such as ‘401 k’, ‘bonus’, and ‘retirement’; the corresponding reviews indicated workplaces in which concerns about long-term financial benefits rather than the presence of implicit incentives in one’s own work were at the forefront.
- *Emotional Aspects* (third set of rows in Table 3). Negative stress companies were associated with words such as ‘horrible’, ‘terrible’, and ‘awful’, confirming, once again, the presence of emotional pressure. Passive companies were instead associated with words such as ‘repetitive’, ‘turnover’, and ‘workload’, confirming the tedious nature of those workplaces. Low stress companies were associated with words such as ‘fair’, ‘friendlygood’, and ‘pays’, and the corresponding reviews described a good work-life balance. Finally, positive stress companies were associated with words such as ‘prestige’, ‘boost’, and ‘reputation’, and their reviews described high performing, dynamic, and fast-paced workplaces.

**Table 3:** Three-word groups present in the reviews of companies of the four stress types. These groups were automatically found by BERTopic and speak to three main workplace characteristics: career drivers, industry and benefits, and emotional aspects. For each group, the top three words are shown together with their normalized word importance. Abbreviations of words describing monetary benefits include pto (paid time off); espp (employee stock purchase plan); hsa (health savings account); 401 k (a retirement savings and investing plan that employers offer).

	Negative stress	Passive	Low stress	Positive stress
Career drivers	Overtime	Vacation	Scheduling	Teamwork
	Mandatory	pto	Flexibility	Supportive
	Shifts	Vacation/sick	Autonomy	Collaborative
Industry or benefits	Discounts	Insurance	401k	Gain
	Sale	Espp	Bonus	Billions
	Coupons	Hsa	Retirement	Software
Emotional aspects	Horrible	Repetitive	Fair	Prestige
	Terrible	Turnover	Friendly/good	Boost
	Awful	Workload	Pays	Reputation

### Evolution of stress types and the Great Recession

**Figure 2:**
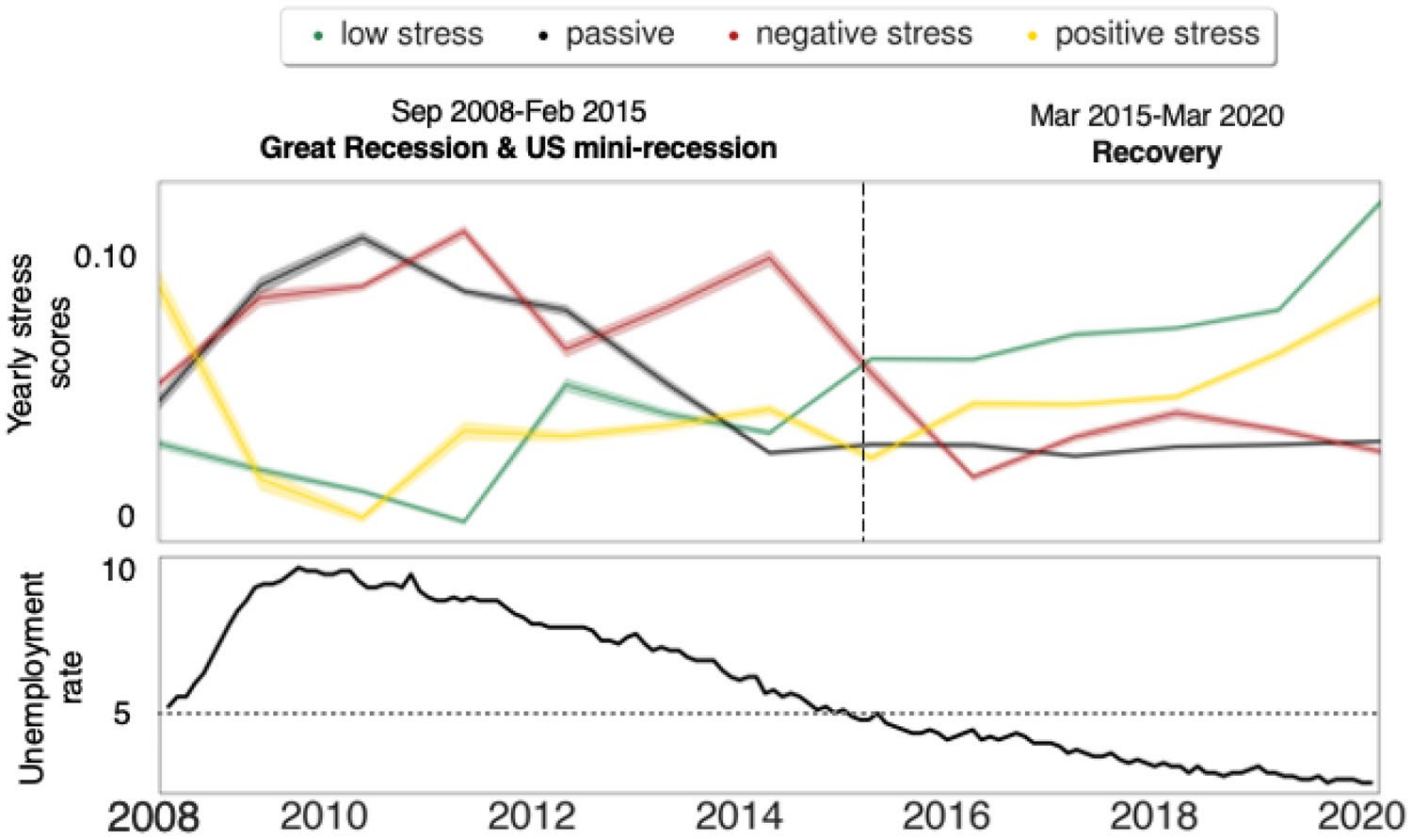
The evolution of: *(top)* the four types of stress; and *(bottom)* the unemployment rate in the U.S., with the horizontal dashed line reflecting pre-recession rate. The stress score per year is calculated using Eq. (2), and its standard deviations are shown with shaded lines.

After the preliminary validation step in which we ascertained that stress was paraphrased in reviews differently according to the stress type, we tested whether the evolution of each stress score over the years tallied with large-scale exogenous events such as the Great Recession. We plotted the amount *m*(*s*, *y*) of each stress score *s* in each year *y* (as per Equation (2)), from 2008 to 2020 (top panel in Fig. 2). The overall changes closely followed the unemployment rates from the U.S. Bureau of Labor (bottom panel in Fig. 2): a year of positive stress (2008) was rapidly followed by several years of negative stress (2009-2015), which peaked during the Great Recession (2009–2011) during which the U.S. went through a loss of over 7.5 million jobs and high unemployment rates^36^.

**Figure 3:**
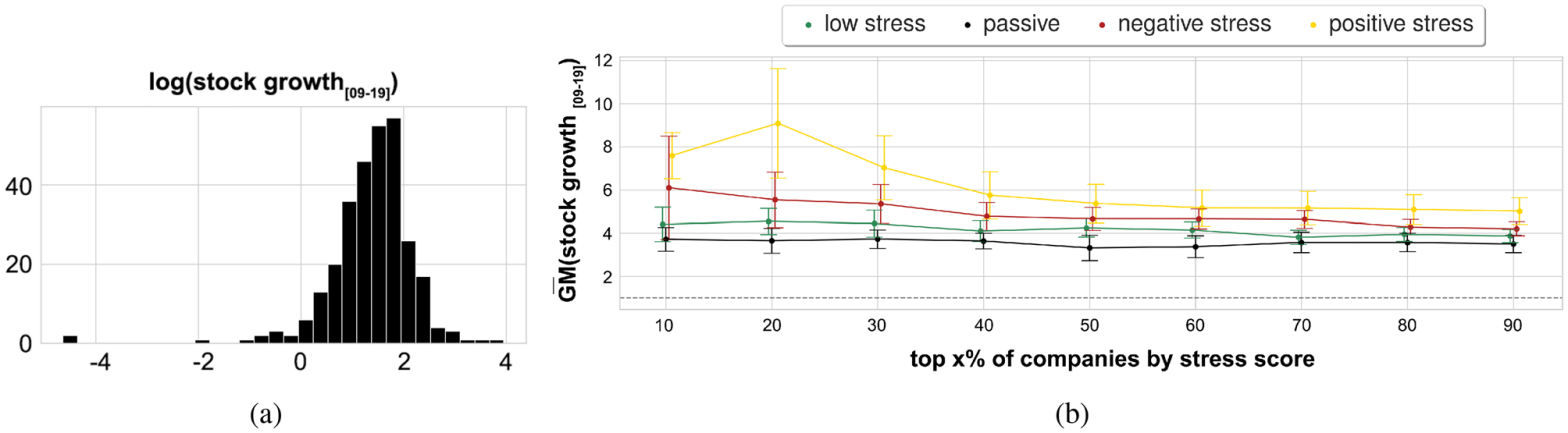
(**a**) Distribution across companies of the logarithm of stock growth values from the average stock price in 2009 and that of 2019 (${stock\_growth}_{[09-19]} = stock_{2019}/stock_{2009}$) showing the stock growth is log-normally distributed. The average stock price for year *y* ($stock_y$) is calculated as the average of the daily Adjusted Closing Prices for the year. (**b**) Geometric mean of the stock growth values ${\bar{GM}}({stock\_growth}_{[09-19]})$ for increasing stress score percentiles for the companies of a given stress type. Error bars represent geometric standard error $GSE({stock\_growth}_{[09-19]}) =$ ${\bar{GM}}({stock\_growth}_{[09-19]})/$ $\sqrt{N} \cdot \sigma (log({stock\_growth}_{[09-19]}))$.

### Stock growth of companies of different stress types

Finally, we hypothesized that a company’s way of dealing with workplace stress was partly *associated* with performance. Given our data, we cannot study whether stress *causes* (poor) performance but can only study how the two are associated. Also, there is no company performance measure that is solely affected by a company’s stress culture. As such, our stress scores are unlikely to be predictive of any company-wide performance measure. We opted for long-term stock growth as our performance measure, not least because it is publicly available and standardized across companies. However, such a growth is partly affected by a company’s culture, and conflates endogenous factors (e.g., productivity) with exogenous ones (e.g., financial cycles). Yet we expected our stress measures to qualitatively describe different forms of financial success, at least in the long term. To that end, we computed stock growth during the full period of study, that is, between 2009 and 2019:

5 $$\begin{aligned} \text {stock growth}_{[09-19]}(c)= \frac{stock(c)_{2019}}{stock(c)_{2009}} \end{aligned}$$

where $$stock^{i}$$ is the average adjusted closing price of company *c*’s stock in year *i*. We chose long-term growth instead of short-term one (e.g., that pertaining 2018-2019) to partly account for any potential influence of exogenous events (e.g., Great Recession, market manipulation, incidental growth/decline^37^). In *Supplementary Information*, we show that the results do not qualitatively change when considering the narrower 5-year period from 2014 to 2019. Given a stress type *s*, we computed company *c*’s *association* *f*(*c*, *s*, *T*) with *s* during time period *T* (initially set to the whole period of study), consequently grouping all the companies of a given stress type into their stress score percentiles (Fig. 3b). As the distribution of stock growth values across companies is heavy-tailed (Fig. 3a), we used the geometric mean to average these values across companies. That is, $$GM({\text {stock growth}_{[09-19]}}) = \Pi (\text {stock growth}_{[09-19]}(c))^{1/n}$$, where *c* is a company in a specific *(stress type,percentile)* bin, and *n* is the number of the companies in such a bin. Positive stress companies enjoyed the highest stock growth with an average value across all percentiles of $${\bar{GM}}(\text {stock growth}_{[09-19]}) = 5.07$$ (Figure 3b), while the average stock growth across the other three types of companies was noticeably lower ($${\bar{GM}}({\text {stock growth}_{09-19}}) = 3.70$$), with passive stress companies exhibiting the lowest growth ($${\bar{GM}}({\text {stock growth}_{09-19}}) = 3.42$$). To ease the interpretation of such values, consider the example of Equinix, a digital infrastructure company headquartered in California, which our approach labeled to be a “positive stress” company. Its stock price traded at 61$ in 2009 and its stock price climbed over 695% (i.e., its $${\bar{GM}}(\text {stock growth}_{[09-19]})$$ was 7.95), trading at 485$ ten years later.

## Discussion

### Limitations

This work has five main limitations. The first concerns the inability of studying whether stress causes performance differences given the absence of cross-lag data that links performance to a stress-related company-wide indicator. Theoretically, we could run a lagged analysis as a linear regression where the dependent variable is the company’s growth at different time intervals. However, such an analysis is hard because of two main reasons: (a) no fine-grained temporal granularity for reviews is possible as reviews might be temporally misaligned since they could be posted after an employee leaves the company, and (b) many, mostly smaller, companies have joined the public reviewing site at later points in time, thus reviews will not cover all 12 years of analysis.

The second limitation is that the decreasing trend of stock growth may be dependent on the two main aspects: company ratings and industry sector. These two have little to do with the hypothesized relationship between stress and performance. We therefore repeated our analyses by considering a company’s overall website rating and its industry sector. As for ratings, we indeed found increasing stock growth with increasing review ratings; still, positive stress companies experienced the highest growth (Fig. S6 in *Supplementary Information*) compared to highly-rated companies. As for industry sectors, we showed that tech companies were over-represented in the positive stress set, and stock growth was partly driven by them (Fig. S7 in *Supplementary Information*). However, by separating companies by industry sector, we still observed that positive stress companies grew more than the other three types (Fig. S8 in *Supplementary Information*).

The third limitation concerns data biases related to temporal granularity and geographic representativeness. Upon new available data, future studies could study workplace stress outside US, allowing for cross-cultural comparisons.

The fourth limitation has to do with nuances when rating a company (e.g., being satisfied with the use of the overall company rating and not its composing dimensions). While on Glassdoor there are several rating fields available, only the overall rating field was mandatory and hence provided sufficient coverage for our analysis.

The fifth limitation is that the deep-learning model used to detect stress mentions in posts is not always accurate. Our medical entity extraction model has two main limitations. First, the model’s strict/relaxed accuracy is .71/.85, and, even though it outperformed competitive baselines by a large margin, it still is not perfect. To partly address this issue, our method limits itself to textual mentions pertaining stress at work. Second, entity extraction models such as ours are not always able to tell apart personal from figurative health mentions (e.g., *‘I felt pain’ vs. ‘He was such a pain to work with’*). This is still an active area of research. Yet our model is relying on a large transformer model (e.g., contextual embeddings RoBERTa), and, as such, it is less likely to make such errors than a simpler, keyword-matching technique. Future studies could use some of the newly published social media datasets^38^ to further train our model to distinguish between different *types of health mentions*.

### Implications

To place our work in the broader context of the literature, we point out three main findings. Our first was that *company reviews contain linguistic markers of four stress types*. Previous work found that stress of social media users can be detected by analyzing their textual content, both on Twitter and Reddit^21^. Another study by Saha and De Choudhury found that high levels of stress markers were present in the use of language in Reddit comments posted by university students who experienced gun violence events at their campuses. This work showed that such linguistic changes are sufficiently subtle to reflect four *different types of stress*, that is, low, passive, positive, and negative stress. Our second finding was that *stress over the years tallied with large-scale exogenous events*. In particular, negative stress was the most prevalent among the four stress types in recession years (both great and mini recessions). This finding is in line with the literature linking economic downturn with stress and mental health issues caused by job instability^39^, and speaks to the presence of linguistic markers reflecting negative stress associated with country-level economic performance. Our third finding was that *company stock growth is associated with positive stress.* This is a new finding, not least because of lack of data in the past. While stock growth conflates endogenous factors (e.g., productivity) with exogenous ones (e.g., financial cycles), we found that positive stress companies enjoyed significantly stronger stock growth.

However, more work is needed to understand how to change a company’s culture into one in which stressors could be used for one’s growth and self-development. Given the recent wave of Great Resignation (i.e., the elevated rate at which U.S. workers have quit their jobs^40^), questions relating to corporate culture^41^ and ways of retaining top talent are of utmost importance. A recent study from Mercer, an American asset management firm, found that elevated levels of employee turnover are not due to lack of engagement at work but attributed to workplace culture and heightened stressors. Therefore, organizations need to take immediate actions by (re)assessing their workplace culture first and by then shifting it when deemed appropriate, through training that fosters psychological safety and cultivates one’s mindset towards positive stress. Traditionally, employee well-being has been tracked with tailored surveys. Automated analyses of the language used by employees on corporate social-networking tools might offer yet another way of tracking workplace stress, which is sufficiently granular to assess the impact of interventions in a company. Beyond the immediate use of these findings for individual companies, several other stakeholders could benefit from our methodology including government officials. As the performance of the S &P 500 companies affects the broader U.S. economy, recommended workplace practices could be established at state- or national-level to improve work conditions.

## Supplementary Information

Supplementary Information.

## Data Availability

We made our code and data available in a readily usable format on GitHub (https://github.com/sanja7s/positive_stress_in_companies) to allow for reproducibility. For each company, we shared the following attributes: company name, #total reviews, #stress reviews, average rating, rating of work-life balance, rating of career prospects, rating of the company, rating of the culture, rating of the management, stress type, strength of association with the stress type, stock values/growth for: 2009, 2012, 2014, 2019, and industry sector.
