# Supplementary material for: Quantifying the impact of positive stress on companies from online employee reviews
Source: Sci Rep. 2023 Jan 28;13:1603. doi: 10.1038/s41598-022-26796-6 (PMC9883817; doi:10.1038/s41598-022-26796-6)
Supplement: Supplementary file 1 — Supplementary Information. [file 41598_2022_26796_MOESM1_ESM.pdf]

Supplementary Information
Quantifying the impact of positive stress on companies from online employee reviews

Sanja Šćepanović[1], Marios Constantinides[1], Daniele Quercia[1,2,*], and Seunghyun Kim[3]

[1]Nokia Bell Labs, Cambridge, United Kingdom
[2]CUSP, Kings College London, United Kingdom
[3]Georgia Tech, USA
[*]quercia@cantab.net

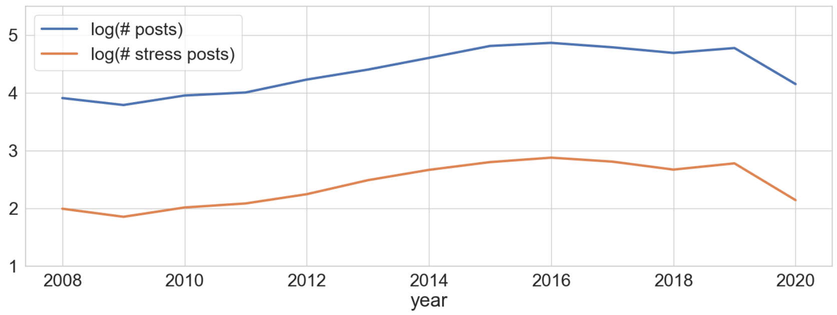

Figure S1. Number of posts (log) in our dataset between 2008 and 2020s. There is no trend difference between all posts and those containing mentions related to stress.

Details of the dataset

We collected a total of 713,018 posts published for a company reviewing site from the start of 2008 up until the first quarter of 2020 for the S&P 500 companies. We filtered out posts belonging to non-US based companies, yielding a total of 439,163 posts across 399 unique S&P 500 companies. The average rating across companies ranged from a minimum value of 1.62 up to a maximum value of 5 ($\mu = 3.37, \sigma = 0.40$). While the overall fraction of stress posts per company was 1.11%, this value ranged from 0% up to 9.52% across companies ($\mu = 1\%, \sigma = 1\%$).

Data representatives

A total of 439,163 posts were analyzed. These posts are about companies distributed across all the 51 U.S. states (Table S1). The highest number of posts were found in California (i.e., a total of 69,968 posts), while the lowest in Wyoming (i.e., a total of 222 posts). The posts span across 11 industries classified according to the Global Industry Classification Standard (GICS), with the highest number of posts for companies in Information Technology, and the least number in Real Estate (Table S2). The posts were written by managers, sales associates, software engineers, analysts, among others (Table S3). Current employees make up 56% of the reviews, and the remaining reviews are predominantly by former employees who held the job within the last five years. The maximum annual number of posts between 2008 and 2020 was observed in 2016, while the lowest number of posts in 2009 (Figure S1).

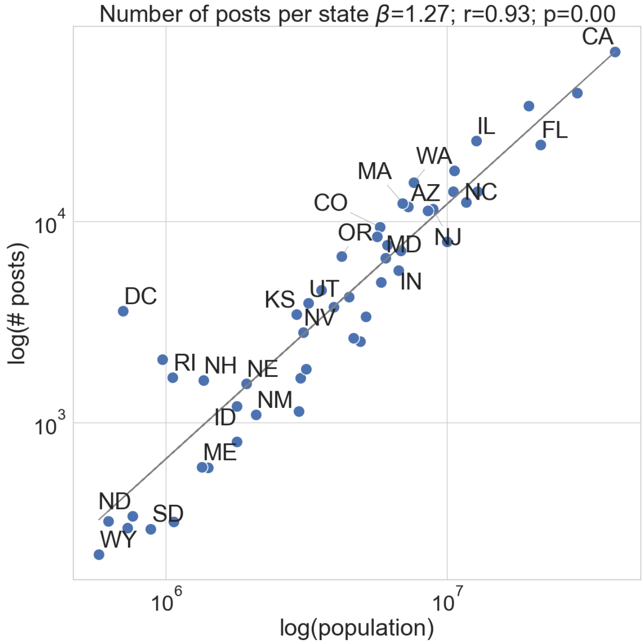

Figure S2. Number of posts (log) in our dataset versus state population (log). The states Washington DC and Rhode Island have more posts that what the population size would suggest. The line of best linear fit is shown in gray. U.S. states are shown with the two-code state abbreviation.

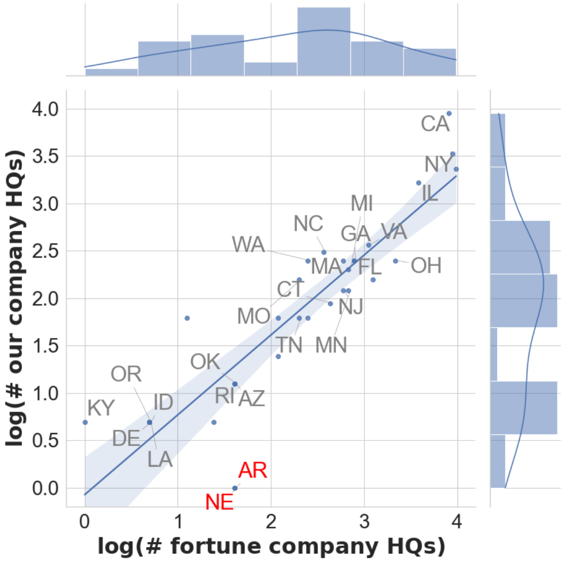

Figure S3. Correlation between the number of headquarters in each state in the Fortune 500 list and the number of headquarters in each state in our dataset (Spearman $r = .90$). The states of Nebraska (NE) and Arizona (AR) have fewer headquarters than what the Fortune list would suggests.

Table S1. Number of posts and number of offices on the company reviewing site across U.S. States, ranked by the number of posts published between 2008 and 2020 in descending order. The state of California had the most published posts, while the state of Wyoming had the least published posts. The Pearson correlation between the log number of posts and the number of companies per state in our data is .98, while the correlation between the log number of posts in our data and the log of population size across states is .93.

U.S. State	# posts	# offices
CA	69968	340
TX	43629	342
NY	37515	313
IL	25157	290
FL	24082	283
GA	17888	275
WA	15672	239
NC	14072	268
PA	14064	271
OH	12447	263
MA	12355	253
AZ	11834	228
NJ	11561	245
VA	11320	235
CO	9408	249
MN	8437	196
MI	7953	237
MO	7657	230
TN	7165	221
OR	6704	206
MD	6610	207
IN	5727	222
WI	5006	181
CT	4567	186
KY	4224	190
UT	3925	187

U.S. State	# posts	# offices
OK	3772	174
DC	3596	180
KS	3459	169
SC	3362	194
NV	2815	163
LA	2631	175
AL	2546	171
DE	2067	113
IA	1849	133
RI	1676	103
AR	1666	149
NH	1627	127
NE	1564	139
ID	1208	112
MS	1138	127
NM	1097	125
WV	803	114
ME	604	105
HI	600	85
ND	344	74
VT	324	54
MT	322	70
AK	300	57
SD	297	57
WY	222	72

Table S2. Number of posts across the Global Industry Classification Standard (GICS) sectors. More posts are generally found in sectors having more companies, as one expects.

GICS Sector	# posts	# companies
Information Technology	63198	52
Consumer Discretionary	62395	40
Financials	49955	42
Health Care	36308	41
Consumer Staples	26471	28
Industrials	24074	43
Communication Services	13842	13
Energy	5510	20
Materials	5269	21
Utilities	3172	19
Real Estate	2228	16

Table S3. Number of posts across roles and statuses.

Employee Title	# posts
Sales Associate	8006
Manager	4536
Software Engineer	4191
Customer Service Representative	4058
Cashier	3726
Director	2819
Project Manager	2365
Senior Manager	2225
Senior Software Engineer	2019
Associate	1969
Store Manager	1963
Assistant Manager	1930
Pharmacy Technician	1747
Analyst	1660
Delivery Driver	1619
**Employee Status**	**# posts**
Current Employee	190876
Former Employee	146449
Former Intern	5207
Former Contractor	3380
Current Intern	2858

Description and evaluation of the deep-learning framework

To extract stress mentions, we used the MedDL entity extraction module[1] (the left rectangle in Figure 1(a)). MedDL uses *contextual embeddings* and a *BiLSTM-CRF sequence labeling architecture*. The BiLSTM-CRF architecture[2] is the deep-learning method commonly employed for accurately extracting entities from text[3,4], and consists of two layers. The first layer is a BiLSMT network (the dashed rectangle in Figure 1(a)), which stands for Bi-directional Long Short-Term Memory (LSTM). The outputs of the BiLSTM are then passed to the second layer: the CRF layer (enclosed in the other dashed rectangle). The predictions of the second layer (the white squares in Figure 1(a)) represent the output of the entity extraction module. To extract the medical entities of symptoms and drug names, BiLSTM-CRF takes as input representations of words (i.e., embeddings). The most commonly used embeddings are Global Vectors for Word Representation (GloVe)[5] and Distributed Representations of Words (word2vec)[6]. However, these do not take into account a word's context. The word 'pressure', for example, could be a stress symptom at the workplace (e.g., *'I felt constant* ***pressure*** *to deliver results'*) or could be used in the physics context (e.g., *'The solid material found in the centre of some planets at extremely high temperature and* ***pressure****'*). To account for context, *contextual embeddings* are generally used. MedDL used the RoBERTa embeddings as it had outperformed several others contextual embeddings, including ELMo, BioBert and Clinical BERT[1].

Our evaluation metric is $F1$ score, which is the harmonic mean of precision P and recall R:

$$F1 = 2\frac{P \cdot R}{P + R} \tag{1}$$

$$P = \frac{\text{#correctly classified medical entities}}{\text{#total entities classified as being medical}}$$

and

$$R = \frac{\text{#correctly classified medical entities}}{\text{#total medical entities}}.$$

For strict F-1 score, we counted as "correctly classified" only the entities that were *exactly* matching the ground truth labels. For relaxed version of F-1 score, partially matching entities are also counted as correctly classified (e.g., if the model extracts the entity "*pain*" given the full mention of "*strong pain*"). Also, given that our data comes with class imbalance (i.e., text

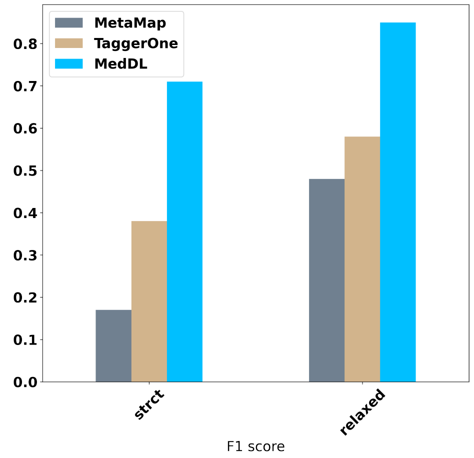

Figure S4. MedDL strict/relaxed F-1 score results when extracting medical symptoms on the MedRed dataset compared to two competitive alternatives of MetaMap and TaggerOne.

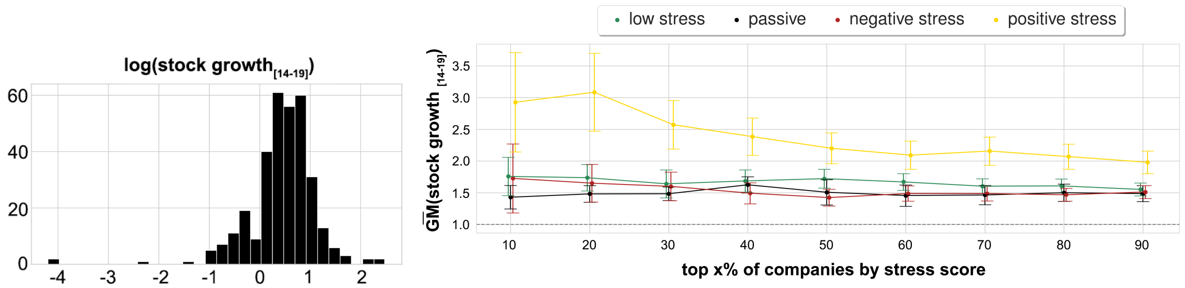

(a) (b)

Figure S5. *(a)* Distribution across companies of the logarithm of stock growth values from the average stock price in 2014 and that of 2019 ($stock\_growth_{[14-19]} = stock_{2019}/stock_{2014}$) showing the stock growth is log-normally distributed. The average stock price for year y ($stock_y$) is calculated as the average of the daily Adjusted Closing Prices for the year. *(b)* Geometric mean of the stock growth values $\bar{GM}(stock\_growth_{[14-19]})$ for increasing stress score percentiles for the companies of a given stress type. Error bars represent geometric standard error $GSE(stock\_growth_{[14-19]}) = \bar{GM}(stock\_growth_{[14-19]})/\sqrt{N} \cdot \sigma(log(stock\_growth_{[14-19]}))$.

tokens do not correspond equally to symptoms, or non-medical entities), we corrected for that by computing P and R using micro-averages[?]. In so doing, we were able to compare Med-DL's F1 scores with those of two well-known entity extraction tools: MetaMap and TaggerOne. MetaMap is a well-established tool for extracting medical concepts from text using symbolic NLP and computational-linguistic techniques[7], and has become a de-facto baseline method for NLP studies related to health[8]. TaggerOne is a machine learning tool using semi-Markov models to jointly perform two tasks: entity extraction and entity normalization. The tool does so using a medical lexicon[9]. The MedDL pre-trained model was evaluated on a labeled dataset of Reddit posts called MedRed. The MedRed dataset was split into train (50%), dev (25%), and test (25%) sets. The MedDL method achieved a strict/relaxed $F1$-score of .71/.85 when extracting symptoms (Figure SI S4), outperforming both MetaMap and TaggerOne by a large margin (the two have $F1$-scores of $.17/.48$ and $.31/.58$, respectively).

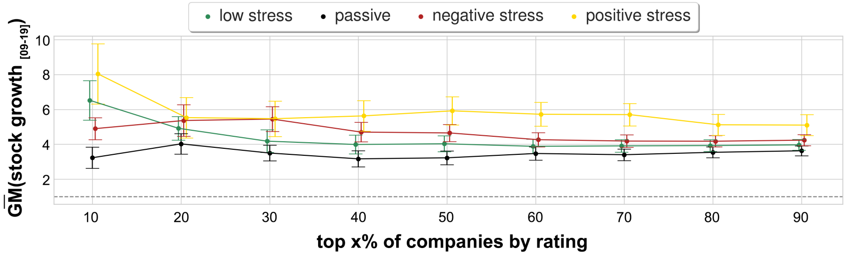

Figure S6. Geometric mean of the stock growth values $\bar{GM}(stock\ growth_{[09,19]})$ for different ratings percentiles for companies of the four stress types. Error bars represent geometric standard error $GSE(stock\ growth_{[09,19]}) = \bar{GM}(stock\ growth_{[09,19]})/\sqrt{N} \cdot \sigma(log(stock\ growth_{[09,19]}))$.

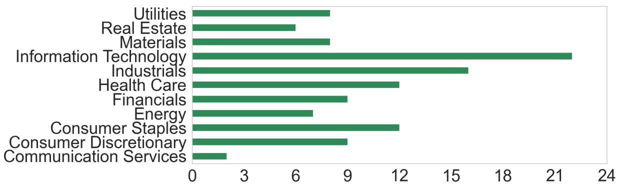

(a) Low stress

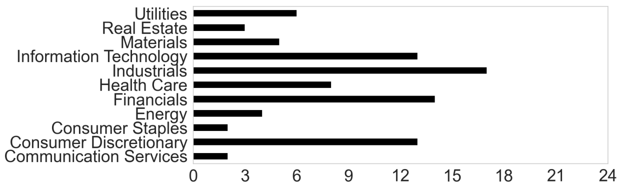

(b) Passive

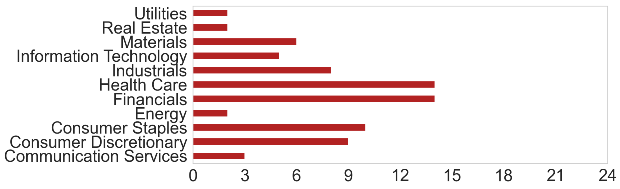

(c) Negative stress

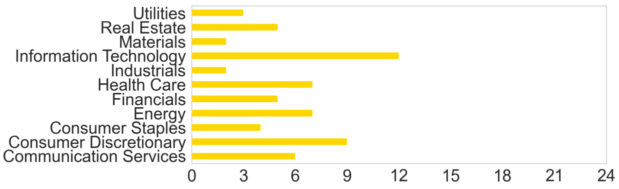

(d) Positive stress

Figure S7. The number of companies per industry sector for the four stress types. IT is more prominent among positive stress companies, while Health Care among negative stress companies.

Annotations of the words BERTopic found. For each topic, we identified the three most representative words and submitted the reviews mentioning them to six annotators. For example, we picked three reviews containing the words ‘overtime’, ‘mandatory’, and ‘shift’ for negative stress companies, and asked six annotators to read them and describe what type of workplaces these reviews would suggest. Upon collecting a total of 72 free-form responses (i.e., each annotator described the reviews corresponding to the 12 topics), we conducted a thematic analysis[10]. To identify overarching themes, we used a combination of open coding and axial coding. We first applied open coding to identify key concepts. Specifically, one of the authors read the responses and marked them with keywords. We then used axial coding to identify relationships between the most frequent keywords to summarize them into semantically cohesive themes.

We found three high-level themes: *career drivers*, *industry or benefits*, and *emotional aspects*. In the reviews, each theme was paraphrased differently depending on the four types of company stress, allowing us to identify sub-themes. The *career drivers* theme described what motivated employees to go to work. Its sub-themes concerned companies whose employees experienced ‘considerable emotional pressure’ (negative stress), tended to ‘focus on activities outside the work’ (passive), cherished ‘their sense of control over their work’ (low stress), and enjoyed ‘a collaborative and supportive workplace culture’ (positive stress). In the *industry or benefits* theme, we identified sub-themes mentioning either the industry sectors of the corresponding companies (e.g., Consumer Discretionary for negative stress, and Information Technology for positive stress) or aspects concerning long-term financial benefits (e.g., passive and low stress). Finally, in the *emotional aspects* theme, we identified sub-themes suggesting employees who experienced ‘emotional pressure’ (negative stress), ‘tedious work’ (passive), ‘good work-life balance’ (low stress), or a ‘fast-paced, high-performing, and dynamic workplace environment’ (positive stress).

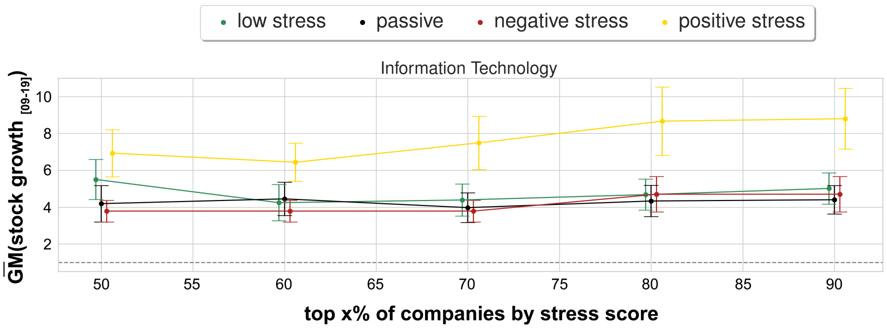

(a)

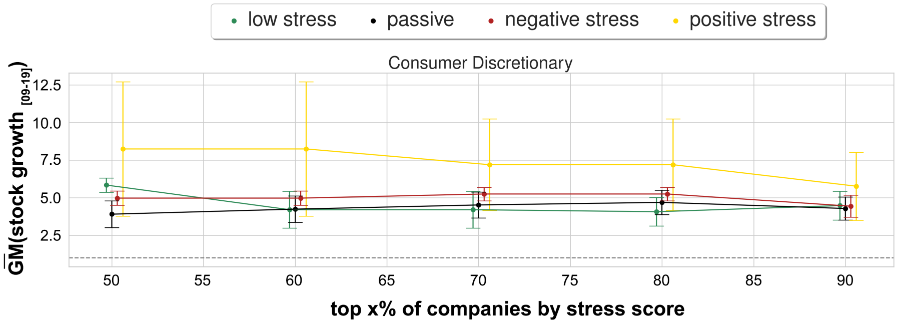

(b)

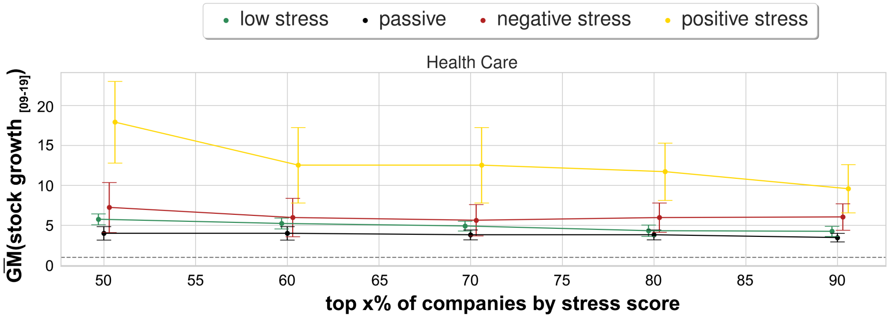

(c)

Figure S8. Geometric mean of the stock growth values $\bar{GM}(stock\_growth_{[09-19]})$ for increasing stress score percentiles for the companies in each of the three most present industry sectors: (a) Information Technology, (b) Consumer Discretionary, and (c) Health Care. The three sectors have sufficient data to ensure statistical significance for each percentile bin. Error bars represent geometric standard error $GSE(stock\_growth_{[09-19]}) = \bar{GM}(stock\_growth_{[09-19]}) / \sqrt{N} \cdot \sigma(log(stock\_growth_{[09-19]}))$.

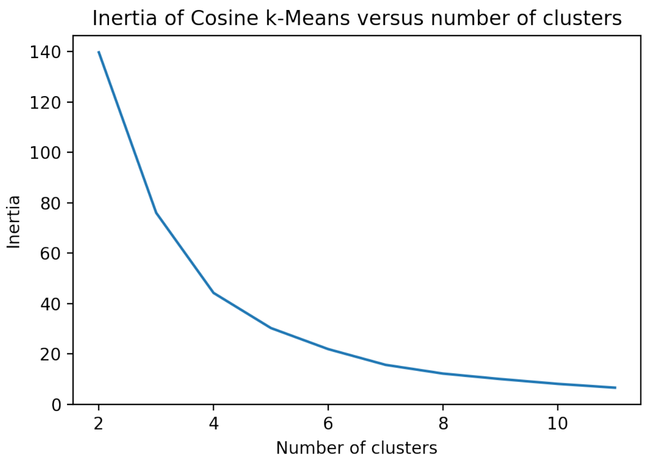

Figure S9. Inertia of Cosine k-Means versus number of clusters having the "elbow" at k=4.

Evaluation of BERTopic results

We ran the topic modeling algorithm BERTopic[11] separately on the four sets of reviews (each set containing reviews of the companies of a given stress type). The fact that BERTopic discovered distinct topics in the four sets reveals that stress is paraphrased differently in the sets. We calculated the topical overlapping values for the different combinations of the four sets (using the Jaccard similarity on the sets of keywords from the top ten topics of each stress type), and found them to be (on average) as low as 0.08 (on a scale ranging from 0 to 1).

Evaluation of the four quadrants

To test whether the quadrant division of companies into four types was meaningful, we manually inspected 30 posts taken at random from companies with high stress, and found stress mentions in companies with low ratings to be qualitatively different from those in companies with high ratings (e.g., a review from a lowly rated company *"The pressure is constantly high, while your work is not appreciated [...] and it feels like the managers do not know what they are doing."* versus a review from a highly rated company *"Happy Employee. Best culture I have experienced, especially in a stressful job. [...] The job is hard, but nothing worth having comes easy."*). Similarly, we found qualitatively different review between companies with low stress and high versus low ratings (e.g., a review from a highly rated company *"Solid company offering Work From Home. [...] decent options to choose for hours worked, great tech support, all equipment supplied, always feel connected to team, strong work ethic. "*, versus a review from a lowly rated company *"Sinking Ship due to Horribly Managed [...] Merger. At legacy X office, they managed to retain some of the positive company culture leftover from the X days. The people are still the best part of that office, but with the increasing turnover, layoffs and "Hunger Games" management style, that is in danger of ending... "*). As a final validity check, we arranged companies along the two axes and clustered them in an unsupervised way. We found four to be the best number of clusters. More specifically, we applied k-means clustering, and searched for the optimal number of clusters using the elbow method (Figure S9). The method involves calculating the sum of squared distances between data points and the k assigned clusters' centroids, for an increasing number of clusters k. Once this value stops decreasing significantly, it means that that the optimal number of clusters is reached.

Sensitivity of the results

Weighting the scores. We explored the effects of weighting the yearly scores in:

$$m(s,y) = \sum_{c \in s} f(c,s,y) \times w(c,y,s), \tag{2}$$

by plotting the temporal scores without weights, i.e., where $w = 1$. The result is shown in Figure S10. The simple aggregation skews the results towards (the long tail of) small companies as it considers a small company equal to a big one.

Shorter-term growth. To test whether our results on stock growth are not affected by exogenous events such as the Great

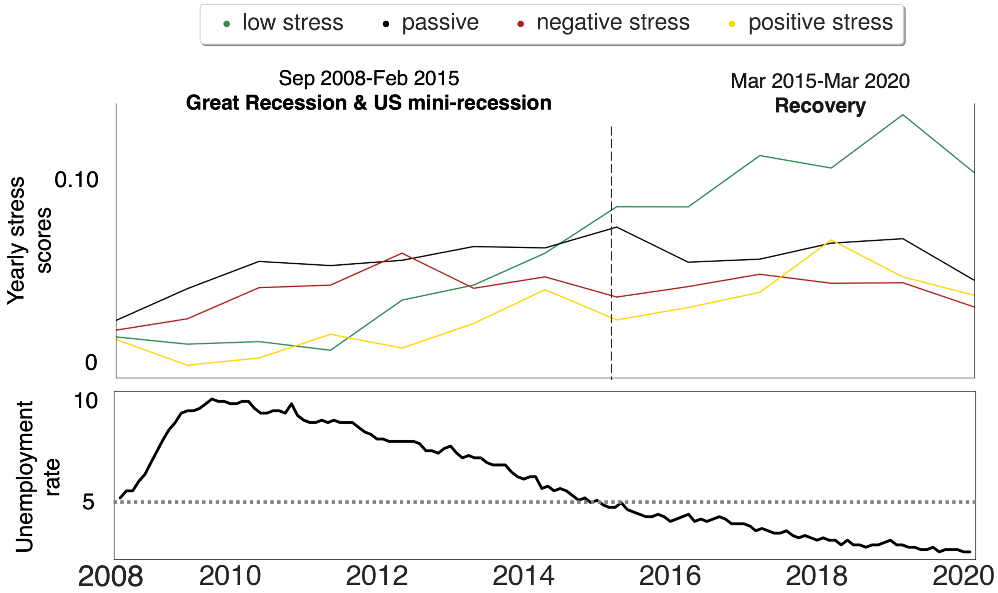

Figure S10. The effects of weighting the yearly scores. *(top)* The evolution of temporal scores without weights, i.e., where $w = 1$ for the four types of stress; and *(bottom)* the unemployment rate in the U.S., with the horizontal dashed line reflecting pre-recession rate. The stress score per year is calculated using Equation (2) with $w = 1$.

Recession, we computed stock growth for the narrower 5-year period between 2014 to 2019:

$$stock\ growth_{[14-19]} = \frac{stock^{2019}}{stock^{2014}} \tag{3}$$

where $stock_i$ is the average adjusted closing price of their stocks in year i. Figure S5 shows that the trend remains qualitatively the same as that in Figure 2, even when removing the Great Recession period. Positive stress companies enjoyed the highest stock growth (with average value across all percentiles being $\bar{GM}(\text{stock growth}_{[14-19]}) = 1.97$ as per Figure S5 on the right), low stress companies had the second highest ($\bar{GM}(\text{stock growth}_{[14-19]}) = 1.53$), while passive and negative stress companies enjoyed the lowest growth ($\bar{GM}(\text{stock growth}_{[14-19]}) = 1.46$, and 1.45, respectively).

Interaction effects between stress scores and review ratings. We tested whether our observed stock growth was genuinely associated with positive stress companies rather than being simply associated with highly-rated companies. To this end, for each stress type, we plotted $\bar{GM}(stock\_growth_{[09-19]})$ against different rating percentiles (Figure S6). Highly rated companies experienced stock growth, yet there are still significant differences across companies of different stress types: in particular, positive stress companies of varying rating percentiles consistently enjoyed the highest growth (the yellow line in Figure S6 is consistently above the other three lines).

Growth per industry sectors. To test whether a specific industry sector is predominant for a given stress type, we first plotted the number of companies per industry sector according to the GICS classification (Figure S7). Information Technology was more prominent among positive stress and low stress companies, Health Care and Financials among negative stress ones, and Industrials and Consumer Discretionary among passive ones. To then check whether the distribution of industry sectors across the four types of stress affected our findings for stock growth, we computed stock growth between 2009 and 2019, and did so for the three most frequent industry sectors separately (i.e., Information Technology, Consumer Discretionary, and Health Care). We chose those three sectors because each individually contained a sufficient number of companies and, as such, allowed us to obtain statistical significant results. Stock growth was computed as $GM(\text{stock growth}_{[09-19]}) = \Pi(\text{stock growth}_{[09-19]}(c))^{1/n}$, where c is each company from a given industry sector (e.g., Information Technology) in a specific *(stress type,percentile)* bin, and n is the number of the companies in such a bin. For the three industry sectors, we plotted $\bar{GM}(stock\_growth)$ against different stress score percentiles (Figure S8). In all three sectors, we observed that positive stress companies had consistently higher stock growth compared to the other three stress types.

Percentage of stress posts. To test the sensitivity of our results to the percentage of stress posts being considered, we repeated

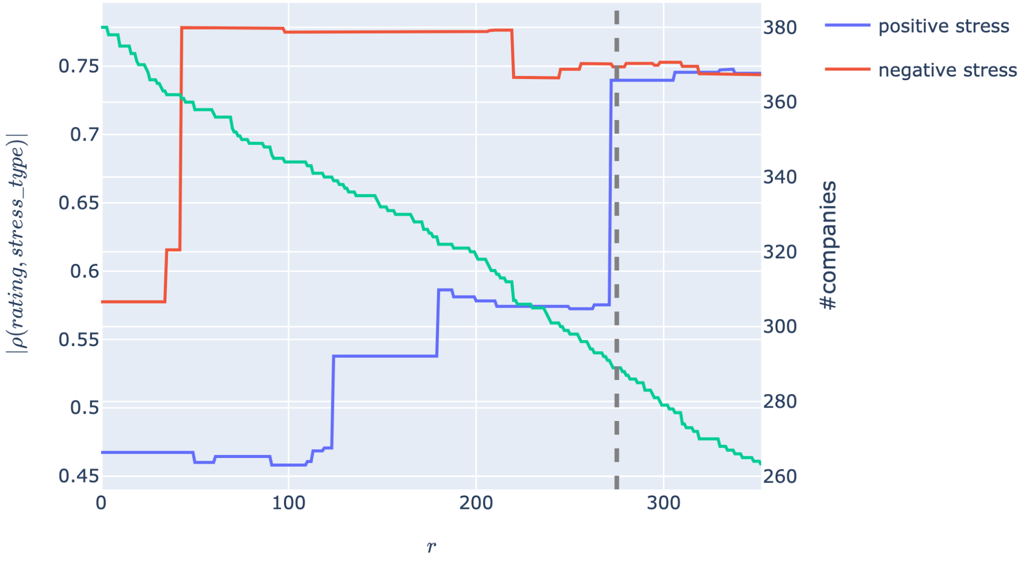

Figure S11. Threshold selection. Correlation values between each of the two stress scores and a company's website overall rating (y-axis) for the companies with at least r reviews (x-axis). These values have a phase shift at $r = 280$ for positive stress companies (blue), matching the value of the correlation for negative stress companies (red).

our analyses by including only the companies with at least r reviews. We found the optimal threshold r to be 280, and did so as follows. To include at least half of the total S&P 500 companies, the least number of reviews per company had to be less than $r = 350$. Then, for each $r = 1, \ldots, 350$, we subset the companies having at least r reviews, and calculated the correlation between a company's rating and its positive stress score (for positive stress companies) or its negative stress score (for negative stress companies), and did so for each subset. We found that the absolute values of the correlations increased with the number of reviews (Figure S11), as one expected, and there was a phase shift at $r = 280$ for positive stress companies (ρ(company_rating, positive_stress_association)=.75). The same applied to negative stress companies (Figure S11). At this threshold, we were left with 287 companies out of 380 companies in total. We repeated the calculations on this subset of companies and, compared to our previously reported results, found even stronger associations between: i) negative stress scores in the whole U.S. and the Great Recession, and ii) a company's positive stress score and its stock growth.

Combining stress and review scores. We fit three Ordinary Least Squares (OLS) models to predict stock growth (Figure S12). In each model, we used the (log of the) number of reviews as a control variable. In addition, (a) M_r uses the average rating score as the additional independent variable (baseline model), (b) M_s uses the stress score, and (c) M_{r+s} uses both the rating score and the stress score as additional independent variables. We applied bootstrapping to ascertain the statistical significance of the results by randomly subsampling a set of 120 companies 10 times. We observed, over the baseline model M_r, a 78% increase of $Adj.\ R^2$ for M_s and a 192% increase for M_{r+s}.

References

1. Šćepanović, S., Martín-López, E., Quercia, D. & Baykaner, K. Extracting medical entities from social media. In *Proceedings of the ACM Conference on Health, Inference, and Learning (CHIL)*, 170–181 (2020).
2. Huang, Z., Xu, W. & Yu, K. Bidirectional lstm-crf models for sequence tagging. *arXiv preprint arXiv:1508.01991* (2015).
3. Straková, J., Straka, M. & Hajic, J. Neural architectures for nested ner through linearization. In *Proceedings of the Conference of the Association for Computational Linguistics*, 5326–5331 (2019).

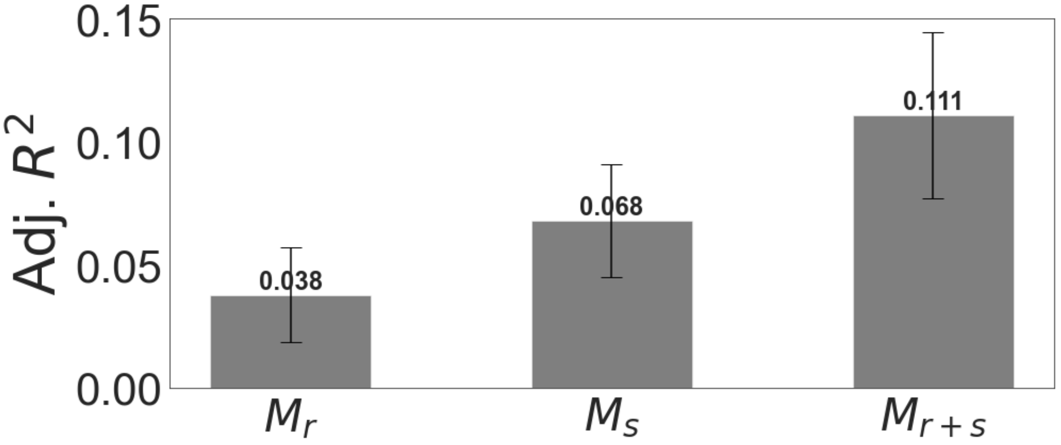

Figure S12. Adjusted R^2 values of three OLS models with different predictors: r is the rating score; s is the stress score; r+s is the rating score and stress score. We applied bootstrapping to ascertain the statistical significance of the results by randomly subsample a set of 120 companies 10 times. Average values and standard deviations are reported. We observed, over the baseline model M_r, a 78% increase of $Adj.\ R^2$ for M_s and a 192% increase for M_{r+s}.

4. Akbik, A., Bergmann, T. & Vollgraf, R. Pooled contextualized embeddings for Named Entity Recognition. In *Proceedings of the Conference of the North American Chapter of the Association for Computational Linguistics: Human Language Technologies, Volume 1*, 724–728 (2019).

5. Pennington, J., Socher, R. & Manning, C. GloVe: Global vectors for word representation. In *Proceedings of the conference on empirical methods in natural language processing*, 1532–1543 (Association for Computational Linguistics, 2014).

6. Mikolov, T., Sutskever, I., Chen, K., Corrado, G. S. & Dean, J. Distributed representations of words and phrases and their compositionality. In *In Proceedings of the Advances in neural information processing systems conference*, 3111–3119 (2013).

7. Aronson, A. R. & Lang, F.-M. An overview of metamap: historical perspective and recent advances. *J. Am. Med. Informatics Assoc.* **17**, 229–236 (2010).

8. Tutubalina, E., Miftahutdinov, Z., Nikolenko, S. & Malykh, V. Medical concept normalization in social media posts with recurrent neural networks. *J. Biomed. Informatics* (2018).

9. Leaman, R. & Lu, Z. Taggerone: joint named entity recognition and normalization with semi-markov models. *Bioinformatics* **32**, 2839–2846 (2016).

10. Braun, V. & Clarke, V. Using thematic analysis in psychology. *Qual. Res. Psychol.* **3**, 77–101 (2006).

11. Grootendorst, M. Bertopic: leveraging bert and c-tf-idf to create easily interpretable topics. *URL https://doi.org/10.5281/zenodo* **4381785**.
